# Gene network requirements for regulation of metabolic gene expression to a desired state

**DOI:** 10.1038/srep01417

**Published:** 2013-03-11

**Authors:** Jan Berkhout, Bas Teusink, Frank J. Bruggeman

**Affiliations:** 1 Systems Bioinformatics, IBIVU, Vrije Universiteit, Amsterdam, The Netherlands; 2 Kluyver Centre for Genomics of Industrial Fermentation/NCSB, The Netherlands; 3 Netherlands Institute for Systems Biology, Amsterdam, The Netherlands; 4 Life Sciences, Centre for Mathematics and Computer Science (CWI), Amsterdam, The Netherlands

## Abstract

Gene circuits that control metabolism should restore metabolic functions upon environmental changes. Whether gene networks are capable of steering metabolism to optimal states is an open question. Here we present a method to identify such optimal gene networks. We show that metabolic network optimisation over a range of environments results in an input-output relationship for the gene network that guarantees optimal metabolic states. Optimal control is possible if the gene network can achieve this input-output relationship. We illustrate our approach with the best-studied regulatory network in yeast, the galactose network. We find that over the entire range of external galactose concentrations, the regulatory network is able to optimally steer galactose metabolism. Only a few gene network parameters affect this optimal regulation. The other parameters can be tuned independently for optimisation of other functions, such as fast and low-noise gene expression. This study highlights gene network plasticity, evolvability, and modular functionality.

Microorganisms are continuously challenged by environmental dynamics to maintain fitness. Sophisticated adaptation mechanisms restore basic cellular functions upon environmental changes^1^,^2^,^3^,^4^,^5^,^6^. These mechanisms invariably involve the sensing and integration of the dynamics of the extra- and intracellular state, and induce adjustments in protein levels through gene expression regulation. In metabolic regulation, dedicated receptors and signalling mechanisms exist only for a few nutrients; generally, the actual state of the metabolic network is sensed by its associated gene network via metabolite-binding transcription factors^7^,^8^,^9^,^10^. On the basis of this information alone, the gene network induces compensatory metabolic gene expression.

Generally, metabolic networks are better understood than their associated gene networks, especially in central metabolism; the stoichiometry and, often, the enzyme kinetics of metabolic reactions are known, or can be determined with existing technologies. However, the identity of the metabolites that regulate the activity of transcription factors of metabolic genes and the kinetics of reactions in the gene network are much harder to determine experimentally. As a consequence, it is not yet understood which metabolic behaviours can be adequately controlled by gene networks and what the functional limits of gene networks are: for instance, can gene networks optimise metabolic functions?

Evolutionary studies indicate that metabolic networks tend to evolve via mutations in their associated gene networks rather than in their metabolic enzyme properties. Laboratory evolution experiments indicate significant adjustments of enzyme levels^11^ and fluxes through metabolic networks^12^,^13^,^14^,^15^ already within hundreds of generations^16^. Remarkably only a few mutations are sufficient, indicating the evolvability and plasticity of gene networks. These studies indicate the importance of gene network control for metabolic functioning and lead to the question whether metabolic functions can be optimised by gene networks to cause considerable increases in fitness. The studies by Dekel et al.^11^ and Ibarra et al.^13^ indicate that gene networks can readily evolve this capability at a single environmental condition, but they do not address whether gene networks can steer metabolism to optimal states over a range of environmental states.

In this paper, we deduce from metabolic information alone the requirement, i.e. the input-output relationship, for the gene network to regulate its target metabolic network in an optimal fashion over a range of environmental conditions. The input-output mapping can be selected on the basis of available data or obtained from a computational, optimization approach. Note that the resulting input-output relationship “mapping” does not have to be unique. After this input-output relationship has been found, relevant questions address whether a given gene network can achieve this behaviour or what candidate gene network structures would be capable of generating the required input-output relationship. Our method can be used in three ways: (i) to parameterise a gene network for which the topology is known but not all the kinetic parameters have been identified, (ii) to identify a (minimal) gene network that is capable of controlling a metabolic system; for instance, by using software to evolve gene network models in the computer^17^,^18^, or (iii) to identify a gene network and metabolic network that both agree with an experimentally determined input-output relationship. We focus in this work on the first application to study the control capabilities of a well-studied gene network.

With the method outlined in this paper, we will study whether the plasticity of a given gene network, for which the topology is known, is large enough to give rise to optimal control of its associated target network. For this we chose the regulation of galactose metabolism in *Saccharomyces cerevisiae*. The galactose network has been studied extensively at the genetic and metabolic level^19^,^20^ and is arguably the best studied regulatory network in this organism. As such, it provides a realistic and relevant network to investigate the interactions between the metabolic and gene-regulatory networks. We find that the gene network is indeed able to optimally steer the metabolic network over a wide range of galactose concentrations. Subsequently, we study whether this trait prevents the gene network from carrying out other functions (optimally), such as fast and low-noise transcription responses. In the case of such restrictions, regulatory trade-offs would occur within the gene network. Interestingly, about one third of the parameters are most important for setting optimal enzyme levels, whereas other parameter sets are more important for regulation upon environmental perturbations, management of molecular noise or avoiding the build-up of toxic metabolic intermediates. Our approach can integrate metabolomics and protein expression data sets and provides a conceptual framework to understand – or engineer – gene regulatory networks that can implement metabolic objectives.

## Results

### Identification of a regulatory gene network for desired metabolic enzyme expression

In this section, we will present the approach for the identification of the properties of a gene network that is capable to steer metabolic gene expression to a desired steady state at different nutrient levels. The approach is not limited to a metabolic network but could address any molecular network and its control system. In later sections, we apply this approach to the galactose network of *S. cerevisiae*.

**Figure 1:**
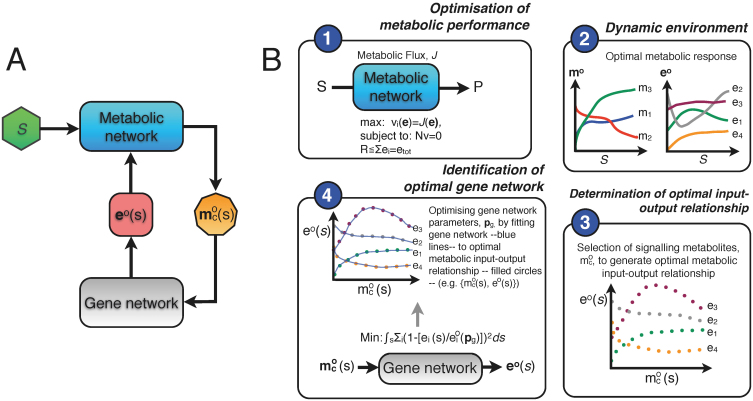
Identification procedure for a regulatory gene network capable to regulate a desired state of metabolic gene expression. (A) Schematic overview of the metabolic and regulatory gene network and their inputs and outputs. Dynamics in the environment, in this example changes in substrate level *s*, lead to altered enzyme expression levels (as indicated by 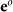) to restore fitness in the perturbed condition. These altered enzyme expression levels are achieved by the regulatory gene network that uses signalling metabolites 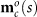 as input. Note that these signalling metabolites are a function of the environmental change. (B) Optimal steering of a metabolic network by a regulatory gene network involves four steps: (1) Optimisation of metabolic performance. The metabolic network is optimised for an objective function under constraints. In this example, optimising the metabolic enzyme levels that lead to the highest steady state flux *J* under the constraint of a limited amount of resource, *R*. (2) The optimisation is performed for different environmental conditions (in this example different nutrient concentrations), yielding the relationship between the external substrate *s* and the optimal metabolite 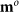 and enzyme 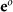 concentrations. (3) From 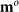 the metabolites signalling to the gene network 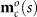 are selected, to form –together with 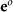
(*s*)– the optimal input-output relationship for the gene network. (4) The gene network receives 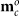 as input and generates 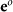 as output. The kinetic parameters of the gene network (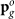) are found by fitting the gene network to the optimal input-output relationship.

The method involves a series of steps that are shown in Figure 1. A mathematical formulation of the procedure is presented in the Methods section. We start from a mathematical model of the metabolic network, typically described in terms of a set of ordinary differential equations and a kinetic characterisation of the enzyme-catalysed reactions. The enzyme levels of this metabolic network will be optimised, given biochemical and evolutionary constraints, for a metabolic objective function. This objective function will typically represent a functional feature of the metabolic network that has a significant contribution to fitness and can be susceptible to natural selection. Below we will restrict ourselves to optimisation of the specific growth rate of *S. cerevisiae*, but the method can deal with other objectives equally well.

The outcome of the optimisation is a vector of optimal enzyme concentrations, denoted by **e*°***, that achieves the optimum and obeys the set of constraints. The optimisation can be carried out over a range of different environmental conditions and therefore we write this optimum as a function of a(n) (environmental) parameter, *s*, i.e. **e*°***(*s*). The parameter *s* will often be the extracellular concentration of the substrate of the metabolic network under consideration or the signal concentration for a signalling network. Substitution of **e*°***(*s*) in the mathematical model for the metabolic network allows for the determination of the steady state metabolite concentrations in the optimal state **m*°***(*s*) (step 2, Figure 1B).

From the vector of optimal metabolite concentrations, we select the concentrations of the signalling metabolites that communicate with the gene network and denote the resulting vector by 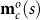, i.e. 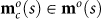 (step 3, Figure 1B). As a result of the metabolic network optimisation, we obtain for every value of *s* corresponding values of optimal levels of signalling metabolites and metabolic enzymes, e.g. 
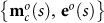
. This information is sufficient for identification of a gene network that can achieve optimal gene expression: if the gene network puts out **e*°***(*s*) given 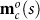 as input, then the metabolic network achieves its optimum at *s*. Therefore, we have to parameterise the gene network in such a way that it acquires as steady state enzyme levels, **e*°***(*s*), when it receives 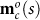 as input. Here we have assumed that *s* does not directly impact the gene network, only indirectly through its metabolic influence on 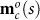; the method can straightforwardly be extended to accommodate this regulatory influence. The identification of the gene network is achieved by parameter fitting of a gene network model (step 4, Figure 1B). The topology of gene networks is generally much better described than their kinetic parameters, therefore we assume that the structure of the gene network is known and we only estimate the kinetic parameters of the gene network. The resulting parameter vector that denotes the best fit of the gene network to the optimal input-output characteristic is denoted by 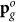.

Rather than fitting the kinetic parameters of a gene network, of which the topology is known, to satisfy the optimal input-output relationship, the method can also be used to find a specific gene network topology and its kinetic parameterisation that can generate optimal input-output relationship. This can for instance be done with algorithms for molecular network evolution^17^,^18^. Here we do not consider this possibility any further but focus on the galactose network in yeast.

### Identification of an optimal gene network input-output relationship for the galactose network in yeast

**Figure 2:**
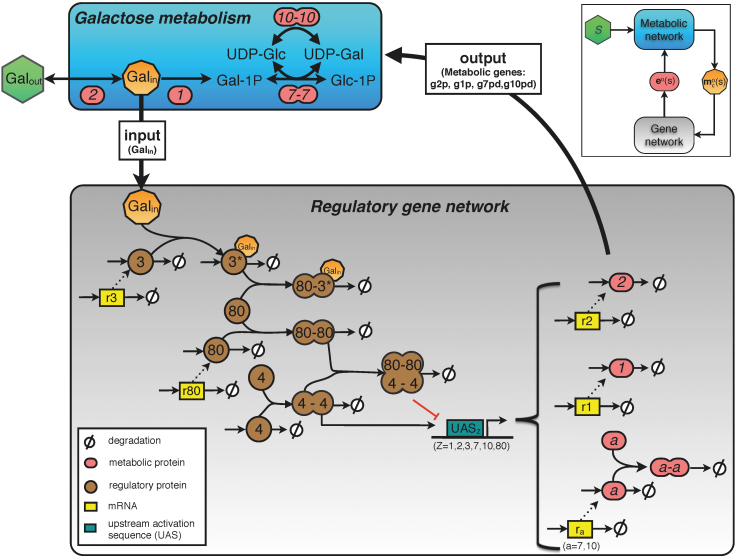
Modular representation of the galactose network and it's regulatory interactions in yeast. Shown are the inputs and outputs of the galactose metabolism and galactose regulatory network, using a similar representation as in Figure 1A. Galactose metabolism (shown in blue) consists of four metabolic enzymes (gal2p, gal1p, gal7pd, gal10pd, shown in red). External galactose (Gal*_out_*, green), is imported by gal2p, resulting in intracellular galactose (Gal*_in_*, orange), which is further metabolised into glucose-1-phosphate (Glc-1P) by the enzymes gal7pd and gal10pd. Gal*_in_* is needed for activation of the galactose regulatory network by binding to gal3p. Within this network, a distinction can be made between the regulatory proteins, gal3p, gal80p, gal4p (brown) and structural proteins (metabolic enzymes; red). Transcription of all genes is dependent on the concentration of gal4p dimer (gal4pd) and the number of gal4dp binding sites that the upstream activating sequences (UAS's) possess. The resulting mRNA's are shown in yellow. Degradation of every mRNA and protein is the net effect of intrinsic degradation and the growth rate dependent dilution.

The metabolic and gene regulatory network of the galactose utilisation system of *S. cerevisiae* has been studied extensively. Detailed mathematical models of the system have been published that capture the existing biochemical information of this complex control system^2^,^21^,^22^,^23^,^24^. The relevant interactions are shown in Figure 2. A description of the mathematical model is given in the Methods section and is based on the model of Atauri et al.^21^.

In a nutshell, the metabolic network considers the following processes. It takes up external galactose (Gal*_out_*), which is in turn converted into glucose-1-phosphate (Glc-1P) by a series of metabolic enzymes. Intracellular galactose (Gal*_in_*) binds to the sensory protein gal3p, leading to its activation. Active gal3p (gal3p*) binds to the repressor, gal80p, thereby preventing the binding of gal80pd to the transcriptional activator gal4pd (‘*d*’, stands for dimer). The protein gal4pd promotes transcription of all *GAL* genes, including the regulatory genes (*GAL80, GAL3*) and structural genes (*GAL2, GAL1, GAL7* and *GAL10*).

**Figure 3:**
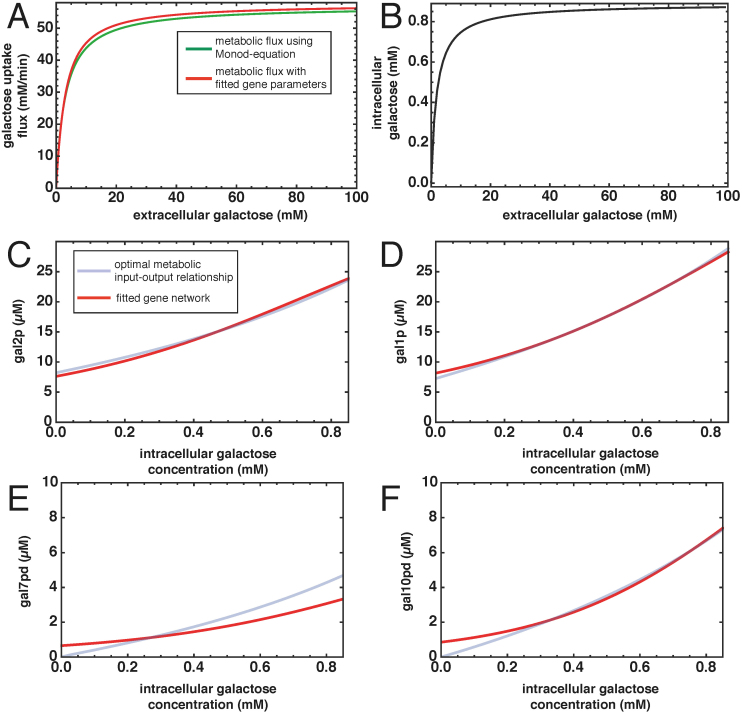
Optimal gene network input-output relationship for the galactose network in yeast. (A) Relationship between external galactose concentration (mM) and the galactose steady state flux (mM/min). The green line corresponds to the galactose flux as obtained by the Monod-equation. The red line shows the metabolic steady state flux that is calculated using the entire galactose model with the fitted gene network parameters. (B) Relationship between environmental dynamics and intracellular signalling metabolite. For a range of external galactose concentrations the corresponding range of intracellular galactose concentrations (the signalling metabolite for the gene network) range between 0 and 0.87 mM. (C-F) Input-output relationship for the galactose gene network. The blue lines correspond to the relationship between intracellular galactose (mM) and the metabolic enzyme concentrations (*μ*M), as obtained by optimising the –isolated– metabolic network. The red solid lines represents the gene network behaviour with the gene kinetic parameters obtained by fitting the gene network to the input-output data. Panels correspond to: C gal2p; D gal1p; E gal7pd; F gal10dp.

We followed the procedure laid out in the previous section (see also Methods). We first performed a constrained optimisation of the metabolic network to identify enzyme levels that optimise metabolic behaviour for a given environment. We consider the case that the specific growth rate of yeast is under selective pressure and is being maximised by adjustments in enzyme levels. This scenario corresponds to a serial-dilution experiment of *S. cerevisiae* continued for hundreds of generations^15^. Translation of this global selective pressure to the level of the metabolic network leads to the objective that the galactose flux per unit protein invested in the metabolic pathway is being maximised. To obtain a relationship between the galactose steady state flux and the external galactose concentration (the environmental parameter), we make use of several microbiological relationships. First, we relate yeast's growth rate to the external galactose concentration using the Monod equation: *μ* = *μ_max_* · [*Gal_out_*]/(*K_s_* + [*Gal_out_*]), with a maximal growth rate, *μ_max_* = 0.4 (hr^–1^) (see Methods and Ref. 25) and a Monod constant, *K_s_* of 3 mM (estimated value). Second, the specific growth rate is related to the galactose uptake rate through the galactose yield: 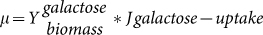. An experimental measured value of 
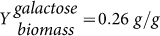
 was used^26^. By using these physiological parameters, the galactose uptake flux ranges from 0.2 to 55 mM/min when the external galactose concentrations varies from 10 *μ*M to 100 mM (green line in Figure 3A). The constrained optimisation of the mathematical model of the galactose metabolic pathway - not coupled to the gene network - involves minimisation of the amount of total protein used in the pathway to reach the galactose uptake flux corresponding to a specific galactose concentration. This procedure is then repeated at discrete intervals over the entire range of external galactose concentrations. We find that for the highest galactose concentration of 100 mM a total amount of 68 *μ*M of total enzyme is minimally needed. Under these conditions, gal1p and gal2p have the highest expression levels of about 30 and 25 *μ*M, respectively and gal7p and gal10p are approximately 7 and 5 *μ*M, respectively (Figure 3C–F). These enzyme amounts vary over the range of environmental conditions.

The regulatory metabolite that communicates between the metabolic and the gene network is intracellular galactose (Figure 2). We find internal galactose concentrations ranging from 0.5 *μ*M to 0.87 mM (Figure 3B) in the optimised metabolic model, which are realistic values given experimental data^15^,^22^,^26^,^27^,^28^. The input-output characterisation of the optimal gene network is obtained by plotting the internal galactose concentration, the metabolic signal to the gene network, versus the optimal enzyme expression levels (blue lines in Figures 3C–F). These dependencies are required for maximal specific galactose uptake fluxes as function of the external galactose conditions. Surprisingly, these dependencies are close to linear relationships. The next question is whether the gene network is capable of generating those dependencies. To address this question, we study the gene network module first in isolation from the metabolic network.

We assumed that the topology of the gene network is known; it is shown in Figure 2. We only allowed the kinetic parameters of this gene network to vary when we fitted this system to the optimal input-output relationship from the metabolic network optimisation (shown in Figure 3C–F). Although we keep the network topology fixed, it should be noted that we do not restrict the values that the kinetic parameters can take. Therefore, some of the interactions in the network can disappear during the fitting procedure. In the fitting procedure we searched for parameter values of the gene network that minimise the squared distance between the desired optimal input-output characteristic and the estimated gene network characteristic. We considered all the four metabolic enzymes curves (Figure 3C–F) simultaneously while fitting. It might well be that other parameter sets lead to an equally good fit. However, here we are mainly interested if we can find such a parameter set in the first place, as this indicates that the current gene network topology is in principle capable of optimally steering galactose metabolism. The resulting optimal gene network parameters are shown in Table S1. We also checked the residuals of the fit and found unstructured patterns, that were almost evenly spread among the four metabolic enzymes (see Supplementary Information, Figure S1). In addition, numerical analysis indicates that the fit indeed found a (local) minimum of the objective function (Supplementary Information, Figure S2).

The panels in Figure 3C–F show the results of the fitting procedure. The fitted gene network approximates the desired input-output relationship very well, except when the concentration of intracellular galactose approaches zero. For the fitted gene network we find that the metabolic enzymes gal7p and gal10p do not go to zero when the galactose concentration drops. This could indicate missing regulatory interactions in the model, such as the shuttling between regulatory proteins between the nucleus and cytosol^23^. However, despite these discrepancies the correspondence of the desired fitness function and the one of the fitted model (Figure 3A) is very satisfactory (maximum deviation is less than 2%, Figure 3A; green and red line).

For growth conditions in batch cultures with galactose medium, the selective pressure acts on the specific growth rate. Experimental findings for this growth condition have shown a constant galactose yield^15^. Following the definition of the yield (*Y* = *μ*/*J*), an increase in specific growth rate follows from an increase in specific galactose uptake rate. An enhanced fitness thus indicates an increase in the galactose steady state flux. This is the reason we have optimized the galactose flux *in silico*. Can this fitted gene network regulate the galactose network to this optimal flux state? We tested whether the network property that natural selection acts upon, the galactose uptake flux, follows the optimal pattern that was prescribed in Figure 3A (green line). Therefore, we calculated the metabolic steady state flux as function of the extracellular galactose concentration using the metabolic network coupled to the optimal gene network. We find a flux profile that nearly overlaps the profile as given by the Monod-equation. Taken together, these results indicate that the galactose regulatory network is indeed capable of optimally controlling the metabolic network over a wide range of external galactose concentrations.

### Regulation of the optimal regulatory gene network

The optimal regulatory gene network brings about changes in enzyme levels to maximise the steady state metabolic flux per unit protein. The required changes in mRNA and protein levels take time to accomplish due to the transcription and translation delays. Note that, even though the steady state behaviour of the network was fitted, the dilution of mRNA and protein by growth is incorporated (dependent on the extracellular galactose concentration; according to the relationship shown in Figure 3A): this makes the time dependency of the system response a variable in the fitting procedure. Adequately-timed responses to environmental perturbations are of crucial importance for cells. This is especially relevant for the galactose network; since the galactose metabolic protein levels can make up to 6–9% of total cellular protein^29^. This makes the protein expression of this network a costly process that should be properly regulated. Therefore, we tested to what extend the optimal regulatory gene network is able to respond dynamically to external perturbations.

**Figure 4:**
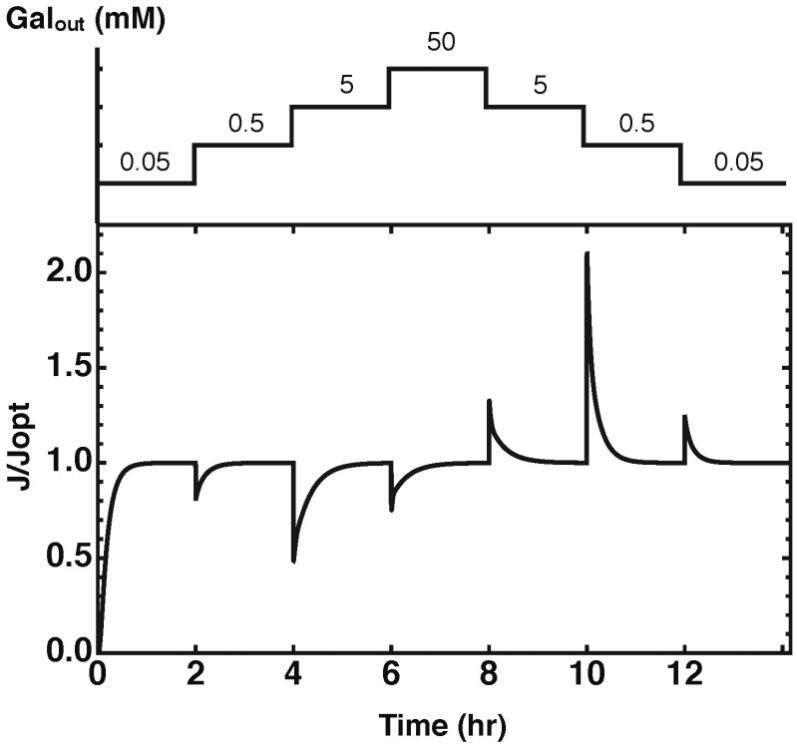
Illustration of optimal tracking of the environment by the optimal regulatory gene network. Shown is the response in the dynamic metabolic flux profile for the model with the optimised gene network parameters with a time interval of two hours between the perturbations. The external galactose concentrations are perturbed as shown in the upper part of the figure and the corresponding response of the metabolic flux is plotted relative to the optimal flux for the indicated galactose concentration.

Thus, we asked whether the gene network, optimised for optimal regulation of the steady state flux, is indeed capable of tracking the dynamic changes in external galactose concentrations and how quickly the optimised model responds. With tracking of dynamic changes we here mean the network's ability to restore the optimal galactose steady state flux upon an external galactose change. If the network is able to restore the steady state flux to an optimal level, as before the perturbation, the network displays perfect adaptation, a well-known concept in systems biology^30^,^31^,^32^. We tested this by perturbing the network with different external galactose concentrations at fixed time intervals of two hours (Figure 4). We found that for the entire range of galactose concentrations considered, the system is able to optimally track the environmental perturbation in this environmental time scale. It always achieves the desired optimal metabolic state and those states are stable. The response times of about one hour are realistic times for yeast.

**Figure 5:**
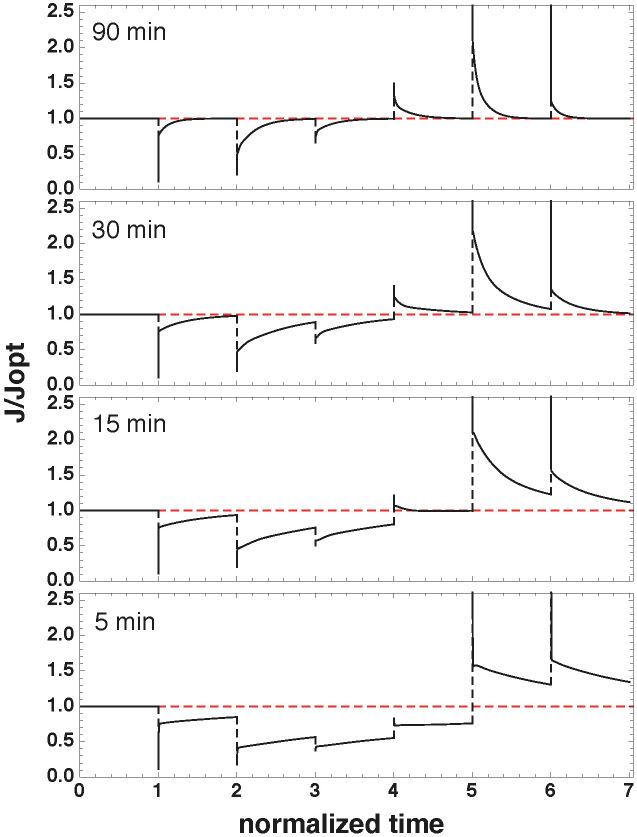
Optimal tracking by the regulatory gene network fails for short switch times. Shown is the metabolic flux profile over time based on metabolic enzyme expression of the gene regulatory network with the fitted gene parameters. The system starts at a steady state with an external galactose concentration of 0.05 mM. External galactose is perturbed in similar steps and using similar concentrations as shown in upper part of Figure 4 at time intervals as indicated in each plot. We plot the metabolic steady state flux relative to the optimal flux at that galactose concentration. The red dashed line corresponds to the optimal metabolic flux for the galactose concentration corresponding to that perturbation. For sake of comparison, we have normalised the time to each perturbation interval, giving rise to the equal space between the perturbations in the different plots.

We explored the dynamic properties of the optimal regulatory gene network by considering shorter time intervals between the consecutive changes in the extracellular galactose concentration. When the time between perturbations is decreased to 90 minutes the optimal gene network can still perfectly track all perturbations (Figure 5). Note that this value is close to the experimental reported maximal response frequency of two strains of *S. cerevisiae* of 1.125 hr^2^. Upon a further reduction in the time between perturbations to 60 and 30 minutes, the optimal tracking capability is lost at the highest and second two highest concentrations, respectively. Time-intervals between the perturbations shorter than about 15 minutes cannot be tracked by the gene network, regardless of the external galactose concentration. This indicates a performance limit of the optimal gene network for steady state tracking.

### Degrees of freedom in the optimal gene network that do not compromise optimal metabolic regulation

After having confirmed that the gene network is capable of optimally steering the metabolic network, we asked whether this optimal behaviour limits the network from carrying out other functions that are potentially relevant for fitness. From the fitting procedure we have obtained a set of parameters for optimal metabolic gene regulation for maximisation of the specific galactose uptake flux. We tested the effect of the fitted parameters on the optimal metabolic regulation by way of a parameter sensitivity analysis: each parameter was perturbed two-fold up or down and the scaled effect on the optimal metabolic flux was calculated.

We deliberately choose to change a 1D parameter sensitivity analysis (changing the parameters one by one) rather than a higher dimensional analysis to investigate cross-dependencies between parameters. We choose this approach because laboratory evolution experiments indicate that generally only one (or two; but typically only one) mutation gives rise to significant fitness changes within hundreds of generations. With this few mutations, parameter cross-dependencies are not of major importance when it is to be determined whether trade-offs between functional traits of the gene network can occur during evolutionary adaptations.

**Figure 6:**
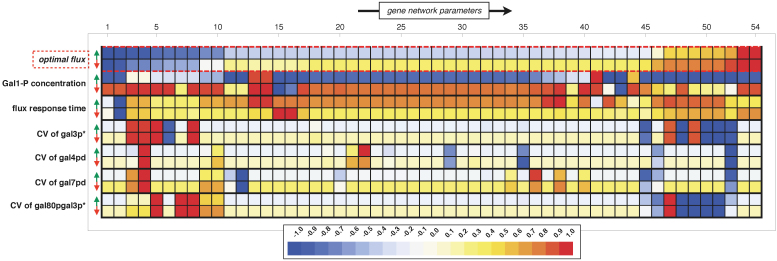
Influence of parameters for optimal metabolic gene regulation by the gene network for other objective functions. Scaled parameter sensitivities corresponding to the fold change in a system property upon 2-fold increase (green arrow) and decrease (red arrow) relative to the unperturbed value. The parameter sensitivities per objective were scaled between -1 and 1 and coloured as indicated by the colourbar. The upper two rows, indicated by the red-dashed box, corresponds to the parameter sensitivities of the optimal metabolic flux and the gene network parameters are sorted according to their influence on this system function. The remaining rows report the effects of the gene network parameters on: the steady state concentration of (potential toxic) metabolic intermediate Gal-1P^27^, the response time of the steady state flux after a shift in the external galactose concentration from 0.5 to 5 mM, and the noise (quantified by the coefficient of variation) calculated from the linear noise approximation^45^ of some key regulators within the gene network: gal3p*, gal4pd, gal7pd and the complex gal80pgal3p*. The numbers above each columns correspond to the gene network parameters as listed in Supplementary Table 1.

In visualisation of the parameter sensitivity analysis results (Figure 6), the effect of all gene network parameters are sorted (and coloured) with respect to this fitness objective (see top two rows in Figure 6). The parameter sensitivity analysis indicates that about 35% (i.e. 19 of the 54) of the parameters have a noteworthy (absolute scaled slope bigger than 0.5) effect on the optimised metabolic flux, whereas the others (35 of the 54) do not affect the flux to a great extend. The parameters that are important correspond to processes affecting the amount of galactose permease (gal2p) as well as the regulatory processes carried out by the proteins gal3p and gal80p. Remarkably, we did not find a great influence of the reactions that involve gal4pd, the exception being the dissociation constant of the gal80pd to the gal4pd-DNA complex. Thus, only a subset of the gene network parameters is important for optimal metabolic regulation.

Next, we asked whether the remaining parameters can be involved in gene network functions that are independent of this optimal metabolic regulation. We performed a parameter sensitivity analysis for three other network objectives relevant for the galactose network: (i) the concentration of metabolic intermediate Gal-1P, which can be toxic to cells; (ii) dynamic response of the metabolic flux to a galactose change and (iii) molecular noise (stochasticity) in several gene network intermediates. In order to make a comparison between the parameter importance among these different scenarios, their values (e.g. scaled parameter sensitivities) were normalised such that they all lie within a range of −1 and 1. Consequently, the relative importance for all parameters for a given scenario can be compared. However, the absolute importance for parameters across scenarios can differ, even though the colours are identical. Each of these scenarios is described below, and shown in Figure 6.

Firstly, we tested which parameters are involved in setting the steady state level of the metabolic intermediate Gal-1P. It has been reported that high concentrations of Gal-1P are toxic in various organisms, including yeast^33^. We found that increasing most of the gene network parameters resulted in reduced Gal-1P concentrations. Exceptions are the intrinsic degradation constants of the complex gal80pgal3p* and gal80p, and dissociation constants for gal4pd binding to metabolic gene *GAL10* and regulatory gene *GAL3* (corresponding parameter numbers are 13, 14, 41 and 44). The first two parameters and the dissociation constant for *GAL3* lead –via their regulatory effects– indirectly to a lower Gal-1P concentration, whereas the consequence of increasing the dissociation constant for *GAL10* acts more directly to the Gal-1P consuming enzymes. For the opposite scenario (two-fold decrease in parameters; red arrow row) a similar pattern emerges: the majority of the parameters results in an increase of the Gal-1P concentration, with again a few understandable exceptions, such as, for instance, parameters that are involved in the Gal-1P producing step.

Secondly, we determined the important parameters for the response time of the galactose network upon a shift in the environmental galactose concentration. Similar to the results in Figure 4 we exposed the network to a galactose upshift from 0.5 to 5 mM. The effect of each parameter in 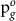 on this dynamic response was quantified by calculating the response-time, e.g. the time required for the system to reach the new optimal flux (see Methods). Metabolic fluxes are the result of many complex interactions (e.g. enzyme and metabolite levels, linkage to other competing fluxes, regulatory networks, etc. see also^34^). This is also reflected by the sensitivity analysis for this scenario: almost all parameters have a substantial effect. Perturbation in the majority of parameters, either up or down, lead to an increase in the response-time of the network. The exception is the degradation rate of gal10p (parameter #2). Note, that this parameter had also the second-highest importance within the optimal metabolic flux.

Finally, the sensitivity of stochastic noise of gene network intermediates to changes in parameters was determined. We have tested the noise of three regulatory proteins, as quantified by their coefficient of variation (standard deviation divided by the mean), and compared this to noise of the metabolic protein gal7pd. Again, we find other parameters that have most effect on stochastic noise, as compared to the optimal flux. The parameters that are involved in the dimerisation reactions and those that affect the concentration of the intermediate of interest are mostly determining the level of noise. Parameter 4 and 52 are important for controlling the noise levels. Interestingly, they both have to do with the central regulator of the network, gal80 (see also^35^). This analysis indicates that different sets of parameters are important for different fitness objectives. This could point at great evolvability of this gene network in a multi-objective evolutionary setting.

## Discussion

Microorganisms exposed to changing environments engage in continuous adaptations of their physiology. Metabolic adaptations typically involve alterations in enzyme expression levels achieved by gene regulatory networks. Currently, a complete systems-level understanding of a molecular network and its underlying regulatory network is often not within reach and generally more information is available for the metabolic network than the gene network. In this study, we presented a method that identifies input-output specifications for a gene regulatory network that can be used either to select candidate gene-regulatory networks or parameterise a network with known topology. This method offers an integrated approach to combine molecular interactions with available experimental data to come to a coherent understanding of regulatory gene networks.

We used this method to study whether the galactose network of yeast is capable of maximising the galactose uptake flux per unit protein. We have shown that the regulatory network is indeed able – by only adjusting it's kinetic parameters – to regulate it's metabolic network to a desired state. However, the proposed method has much wider applicability than considered in this work. For instance, the input-output relationship does not need to be an optimal relationship. In fact, any relationship between input and output can be used, such as captured by experimental proteomic and metabolomics data. In addition, the method is not restricted to metabolic-gene network interactions but applies to any two networks where one carries out a function and the other acts as the controller. Alternatively, the gene network topology can be allowed to vary in the optimisation procedure. In contrast to a fixed topology as used in this study, it would be of particularly interest to incorporate unstructured networks into the approach. The use of an input-output approach together with the modular analysis of hierarchical networks offers interesting possibilities for finding candidate optimal network structures^36^,^37^,^38^. In addition, *in silico* network evolution algorithms^17^,^18^ can be used to find (minimal) network architectures and parameterisations leading to optimal input-output relationships.

One of the surprising findings of this work is that the optimal input-output relationship for the gene network turns out to be nearly a linear relationship (Figure 3C–F). Yet, the regulatory gene network appears much more complex than required for achieving this linear input-output characteristic and may suggest redundancy and multi-tasking (Figure 2). The parameter sensitivity analysis (Figure 6) indicates that this complexity can serve a function in biological systems; it may allow distributed parameter sensitivity over different fitness contributing functions. Different functions of the gene network seem to be tuned independently by alternative sets of parameters. With the sensitivity analysis we aimed to unravel the different parameter (sets) responsible for different network functionalities. From an evolutionary viewpoint, new functionalities can be brought about by the introduction of mutations. Genome wide studies have shown that the accumulation of mutations is a rare event. Simultaneous perturbations of multiple parameters thus seems evolutionarily unlikely. Therefore, we have restricted ourselves to single parameter perturbations and not considered pairs or multiple combination of parameters.

The finding of the different parameter sets, each responsible for a different gene network behaviour, could facilitate the evolvability of molecular control systems as it suggests that trade-offs are unlikely. The decoupling, together with the modular nature of this regulatory network, are two fundamental properties that give rise to robust regulatory systems^39^. Modular networks can maintain advanced network functions due to the strong coupling (high level of complexity) within each level, whilst these levels are connected via only a couple of regulatory interactions. Consequently, and in line with our results, the galactose network displays a key feature of biological systems: large changes in fitness are possible as a result of only a few mutations. Moreover, the fact that galactose metabolic enzymes can make up to almost 10% of total cellular protein^29^, signifies why such tight regulation of the galactose network is important for yeast cells^20^.

In light of the evolutionary history of yeast, the above “decoupling” result also suggests that the regulatory network of the galactose network has been exposed to multiple objective functions during evolution. Depending on the environmental conditions (one can think of, for instance, the availability of multiple carbon sources), other objective functions than considered in this work, could be important. This is in agreement with experimental observations from multiple organisms, for which it was shown that metabolism operates close to the boundary of a solution space defined by multiple (competing) objective functions^40^,^41^,^42^.

This work also indicates the slow response-time of gene networks as a likely limitation of their functioning (Figure 5), which was also found experimentally in yeast^2^. Typically, the response-time of a molecular network scales with the degradation-rate of all the proteins, leading to an interesting trade-off that is hard to overcome by cells: fast response-times require short-lived proteins that can be achieved either by targeted degradation mechanisms or high protein turnover at steady state, which is energetically costly and likely fitness decreasing if organisms compete for growth rate or biomass yield. It is unclear at this stage how cells cope with this trade-off; whether they have evolved for small changes in enzyme levels or whether they prefer suboptimal states to prepare for future changes in environments.

## Methods

### Description of the galactose network in yeast

#### Metabolic network

The metabolic network consists of four metabolic enzymes: galactose permease (gal2p), galactokinase (EC 2.7.1.6, gal1p), galactose-1-phosphate uridylyltransferase (EC 2.7.7.12, gal7p) and UDP-galactose 4-epimerase (EC 5.1.3.2, gal10p). Gal2p is involved in the transport of extracellular galactose (Gal*_out_*) inside the cell, resulting in intracellular galactose (Gal*_in_*). Gal*_in_* is phosphorylated by gal1p yielding galactose-1-phosphate (Gal-1P). Gal-1P is converted into glucose-1-phosphate (Glc-1P) by the action of the two dimeric proteins gal7pd and gal10pd using a UDP-moiety as co-substrates.

#### Transcription and translation

The level of transcription induction is dependent on galactose induction via the sensorial protein gal3p, but also depends on the number of binding sites for gal4pd. The number of gal4pd binding sites assumed in the model are (based on^21^): one for *GAL3* and *GAL80*, two for *GAL7*, four for *GAL1* and *GAL10* and five binding sites for *GAL2*. The degradation rate, for genes and proteins, is the net result of two components: intrinsic degradation and dilution due to cell growth.

#### Control network

The control network is based on the following regulatory interactions: (i) gal4p binds to DNA as a dimer (gal4pd); (ii) gal80p dimerises (gal80pd) and forms a complex with gal4pd; (iii) gal3p binds to Gal*_in_* and forms active gal3p (gal3p*); (iv) gal3p* and gal80p form a complex. Overall, resulting in a decrease in the gal80pd when galactose concentration increases.

### Description of the mathematical model

The mathematical equations of the model are based on Atauri et al.^21^. The unit for concentration is molecules per cell (m/c); to convert from m/c to mM we used 2.38 mL of cell volume per gram of cell dry weight^26^ and a cell dry weight of 15 × 10^−12^ gram per haploid cell^43^. The following minor modifications to the model have been made:

- The reaction catalysed by galactokinase has been shown to be inhibited by it's product, Gal-1P^44^, this was implemented using the rate equation: 
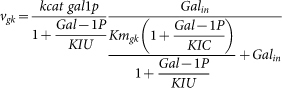
, with KIU = 19.1 (mM); KIC = 160 (mM) as reported by^22^.
- To simulate the environmental perturbations, we have varied external galactose from 0.01 to 100 mM. This wide range has some implications for other model properties, such as the growth rate and degradation rates. To account for these, we have made the degradation rate growth rate dependent. This was done using the Monod equation, relating the growth rate, *μ* (hr^−1^) to the external galactose concentration (mM), with a Monod-constant, *K_s_* = 3 mM (estimated value) and *μ_max_* = 0.4 (hr^−1^). The latter was based on^25^ who reported values of 0.47 and 0.4 (hr^−1^) for the yeast strains *S. cerevisiae* BY4716 and RM11-1a growing on galactose, respectively.
- The degradation rates of all genes and proteins in the model are equal to the summation of two components: intrinsic degradation of the corresponding RNA and proteins, and the other the dilution rate that accounts for cell growth. The latter process we have made galactose dependent using the Monod-equation.
- In the original model, within the control network, two subclasses are defined: control and structural variables. The genes (and corresponding proteins) *GAL3* and *GAL80* are classified as control genes (or control proteins). And *GAL2*, *GAL1*, *GAL7* and *GAL10* genes (or proteins) are considered as structural genes (or structural proteins). Originally, a single parameter value describing the rates of degradation, initiation or the binding affinity of gal4pd to the promoters were used for the entire subclass. Here, we have introduced separate parameters for every single reaction in the regulatory gene network. Resulting in a total number of 54 parameters in the gene network, that were used as variables in the fitting procedure (see Supplementary Table 1).
- We have not taken cooperative binding of genes with multiple binding sites for gal4pd into account.

#### Optimisation of the metabolic pathway

We start from a mathematical model of the metabolic network in terms of ordinary differential equations: 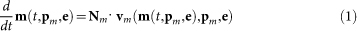
The vector **m** contains the concentrations of the metabolic intermediates and *t* denotes time. The vector **p***_m_* is the parameter vector that contains all kinetic parameters and a characterisation of the environment. The matrix **N***_m_* and the vector **v***_m_* denote the stoichiometric matrix and the rate vector of the metabolic network, respectively. The rate vector contains the kinetic description of all the *r* metabolic reactions in terms of (enzyme) rate equations deriving from biochemistry, e.g. a Michaelis-Menten equation. The vector **e** contains the enzyme concentrations. We consider the metabolic network at steady state: 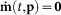. Next, we perform a constrained optimisation of metabolic network performance: 
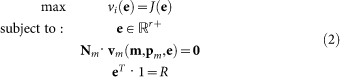
Or, equivalently, 
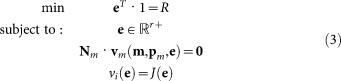


#### Fitting of the gene network

The outcome of these optimisation problems is a vector of enzyme concentrations **e*°*** that achieves the optimum. *R* denotes the total protein (resource) concentration. As we will perform these optimisations over a range of different environmental conditions, we will write this optimum as a function of a parameter, 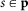, i.e. **e°**(*s*) and 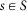. Substitution of **e°**(*s*) in the steady state condition for the metabolic network allows for the determination of the metabolite concentrations in the optimal state, **m°**(*s*); i.e. by solving **N***_m_* · **v***_m_*(**m°**(*s*), **p**, **e°**(*s*)) = **0** for **m*°***(*s*). From the vector of optimal metabolite concentrations we select the concentrations of the signalling metabolites that communicate with the gene network and denote the resulting vector by 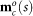, i.e. 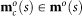. As a result of the metabolic network optimisation we obtain for every value of *s* the tuple 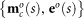. The identification of the gene network is achieved by parameter fitting of a gene network model. The gene network model is described in terms of ordinary differential equations, 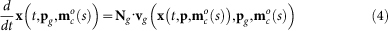
The vector **x** denotes the concentrations of all the intermediates in the gene network, e.g. concentrations of transcription factors, mRNA and proteins. The metabolic enzymes are either all the produced proteins by the gene network or only a subset of them. Hence, **e°** is component (sub vector) of **x**. The identification of the gene network can be expressed as the following optimisation problem where kinetic parameters of the gene network, **p***_g_*, are being optimised, 
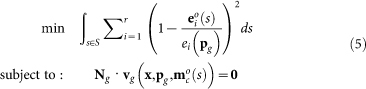
Hence, we here assume that the structure of the gene network is known and we only estimate kinetic parameters of the gene network. The method can in principle also be extended to identify gene network structure that achieves optimal metabolic gene expression. Note that the integration over 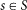 will often be carried out as a sum over instances in *S*. We will denote the parameter vector that denotes the best fit by 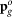.

The entire dynamic system where the metabolic and gene network are coupled is given by, 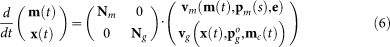
Here we made the occurrence of the environmental parameter *s* in the parameter **p***_m_* explicit.

#### Sensitivity analysis

In the sensitivity analysis all parameters of vector 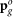 are varied two-fold up and down. The scaled slope of the effect of perturbing gene parameter *p*, on gene network function, *f*, was calculated according to 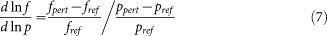
where ‘*ref*’ corresponds to the unperturbed scenario, and ‘*pert*’ corresponds to the perturbation. To allow for comparison of parameter importance for different gene network functions we re-scaled all the slopes as calculated with equation 7, such that their minimum and maximum values lie between −1 and 1 and plotted them accordingly to a colour scheme as shown in Figure 6. For all gene network functions an external galactose concentration of 5 mM was used, except for the flux response time, here the external galactose concentration was increased from 0.5 to 5 mM.

#### Gene network functions

The response time of the metabolic steady state flux upon a perturbation in external galactose was quantified by calculating the time-constant *τ* (unit: time). To calculate *τ*, the response function of the relative steady state flux, was fitted to the function: 1 − *αe*^−*t*/*τ*^, where, *t* stands for time, *α* is a dimensionless parameter (equal to *J*(*t* = ∞)/*J*(*t* = 0); not further used in our analysis) and *τ* represents the response-time constant for which we calculate the parameter sensitivities.

The coefficient of variation (CV) is taken as a measure for noise in molecular species. The CVs were calculated using the linear-noise approximation (LNA). LNA assumes a Gaussian distribution for the probability density function of the molecular numbers at steady state (〈**n**〉*_S_*). In steady state LNA, the covariance matrix 〈*δ***n***δ***n**〉 can be derived from the following fluctuation dissipation theorem^45^, 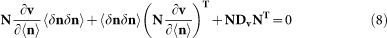


It contains the Jacobian matrix 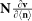, the rate vector **v** and the stoichiometric matrix **N**. A diagonal matrix is denoted by **D_v_**, with the elements of vector **v** as diagonal elements. The CV of species *X* is defined as 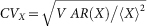, where *V AR*(*X*) is the variance of species *X* as calculated using equation 8 and 〈*X*〉 is the mean concentration of *X* which is calculated by solving the system of ordinary differential equations.

## Author Contributions

J.B., F.J.B. performed experiments. J.B., B.T., F.J.B. analysed data and wrote the paper.

## Supplementary Material

Supplementary Information Supplementary Info
