# Supplementary material for: Gene network requirements for regulation of metabolic gene expression to a desired state
Source: Sci Rep. 2013 Mar 11;3:1417. doi: 10.1038/srep01417 (PMC3593220; doi:10.1038/srep01417)
Supplement: Supplementary Information — Supplementary Info [file srep01417-s1.pdf]

Gene network requirements and limits for regulating metabolic gene expression to a desired state

Jan Berkhout[1,2], Bas Teusink[1,2,3], Frank J. Bruggeman[1,3,4,*]

1. Systems Bioinformatics, IBIVU, Vrije Universiteit, Amsterdam, The Netherlands

2. Kluyver Centre for Genomics of Industrial Fermentation, The Netherlands

3. Netherlands Institute for Systems Biology, Amsterdam, The Netherlands

4. Life Sciences, Centre for Mathematics and Computer Science (CWI), Amsterdam, The Netherlands

* Corresponding author: f.j.bruggeman@vu.nl

Supplementary Figure 1

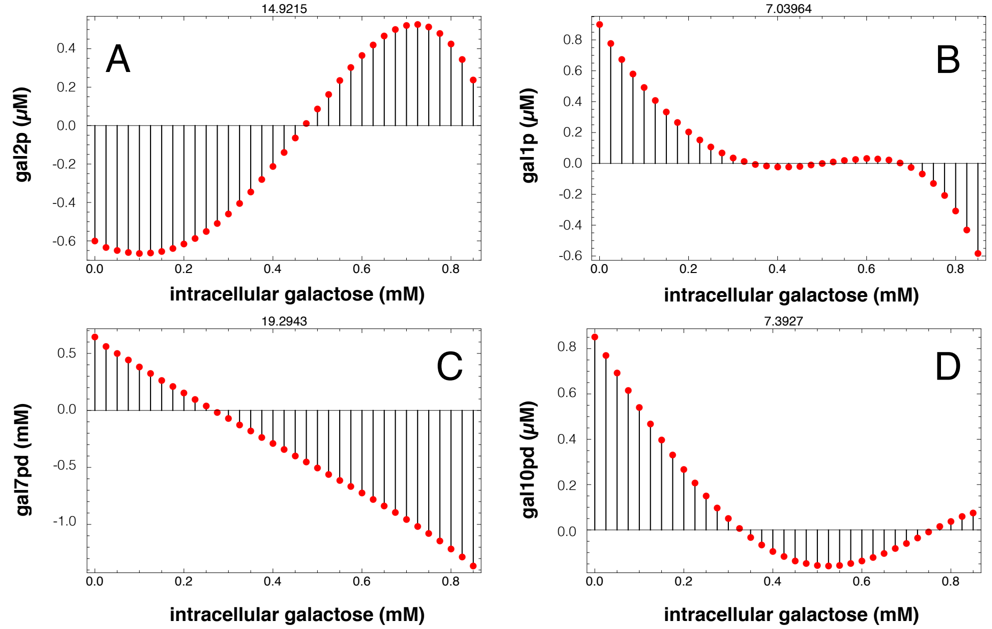

Figure S1: **Residuals of the gene network fit**. Shown are the residuals, in μM, for the four metabolic enzymes as function of intracellular galactose. The number on top of each graph represents the sum over all internal galactose concentration of all residuals for that enzyme. A gal2p; B gal1p; C gal7pd; D gal10dp.

Supplementary Table 1

Table S1: Fitted parameter values of the gene network.

parameter	description	value	unit	# in Fig. 6
idg10d	intrinsic degradation constant of gal10pd	1.851 x 10^{-1}	min^{-1}	37
idg10	intrinsic degradation constant of gal10p	5.150 x 10^{-1}	min^{-1}	44
idg1	intrinsic degradation constant of gal1p	2.650 x 10^{-1}	min^{-1}	42
idg2	intrinsic degradation constant of gal2p	2.727 x 10^{-1}	min^{-1}	1
idg3i	intrinsic degradation constant of gal3p*	1.010	min^{-1}	18
idg3	intrinsic degradation constant of gal3p	1.065	min^{-1}	8
idg4dg80d	intrinsic degradation constant of gal4pd-gal80pd complex	1.537	min^{-1}	22
idg4d	intrinsic degradation constant of gal4pd	1.050	min^{-1}	23
idg4	intrinsic degradation constant of gal4p	1.167	min^{-1}	19
idg7d	intrinsic degradation constant of gal7pd	2.952 x 10^{-1}	min^{-1}	38
idg7	intrinsic degradation constant of gal7p	2.252	min^{-1}	36
idg80d	intrinsic degradation constant of gal80pd	2.518	min^{-1}	32
idg80g3i	intrinsic degradation constant of gal80p-gal3p* complex	1.331	min^{-1}	47
idg80	intrinsic degradation constant of gal80p	1.996	min^{-1}	45
idr10	intrinsic degradation constant of *GAL10*	5.997	min^{-1}	41
idr1	intrinsic degradation constant of *GAL1*	4.889	min^{-1}	43
idr2	intrinsic degradation constant of *GAL2*	9.803 x 10^{-2}	min^{-1}	2
idr3	intrinsic degradation constant of *GAL3*	2.549 x 10^{1}	min^{-1}	5
idr7	intrinsic degradation constant of *GAL7*	5.984	min^{-1}	40
idr80	intrinsic degradation constant of *GAL80*	8.584 x 10^{1}	min^{-1}	52
kfvg3i	association rate constant of Gal_{in}-gal3p complex	2.788 x 10^{-4}	$(m/c)^{-1}$ min^{-1}	48
kfvg10d	association rate constant of gal10p-gal10p dimer	1.835 x 10^{-1}	$(m/c)^{-1}$ min^{-1}	17
kfvg4dg80d	association rate constant of Gal_{in}-gal3p complex	1.505 x 10^{2}	$(m/c)^{-1}$ min^{-1}	21
kfvg4d	association rate constant of gal4p-gal4p dimer	2.163 x 10^{1}	$(m/c)^{-1}$ min^{-1}	31
kfvg7d	association rate constant of gal7p-gal7p dimer	4.729	$(m/c)^{-1}$ min^{-1}	20
kfvg80d	association rate constant of gal80p-gal80p dimer	9.641	$(m/c)^{-1}$ min^{-1}	10
kfvg80g3i	association rate constant of gal80p-gal3p* complex	9.353	$(m/c)^{-1}$ min^{-1}	49
kipg2	maximum initiation rate constant of gal2p	8.698 x 10^{2}	$(m/c)^{-1}$ min^{-1}	53
kipg3	maximum initiation rate constant of gal3p	4.419 x 10^{3}	$(m/c)^{-1}$ min^{-1}	50
kipg4	maximum initiation rate constant of gal4p	1.508 x 10^{1}	$(m/c)^{-1}$ min^{-1}	35
kipg80	maximum initiation rate constant of gal80p	3.117 x 10^{3}	$(m/c)^{-1}$ min^{-1}	4
kipstructg10	maximum initiation rate constant of gal10p	1.216 x 10^{4}	$(m/c)^{-1}$ min^{-1}	16
kipstructg1	maximum initiation rate constant of gal1p	1..398 x 10^{4}	$(m/c)^{-1}$ min^{-1}	13
kipstructg7	maximum initiation rate constant of gal7p	1.456 x 10^{4}	$(m/c)^{-1}$ min^{-1}	11
kir2	maximum initiation rate constant of *GAL2*	3.666 x 10^{1}	$(m/c)^{-1}$ min^{-1}	54
kir3	maximum initiation rate constant of *GAL3*	7.819 x 10^{2}	$(m/c)^{-1}$ min^{-1}	51
kir80	maximum initiation rate constant of *GAL80*	2.392 x 10^{2}	$(m/c)^{-1}$ min^{-1}	3
kirstructr10	maximum initiation rate constant of *GAL10*	3.154 x 10^{2}	$(m/c)^{-1}$ min^{-1}	15
kirstructr1	maximum initiation rate constant of *GAL1*	1.468 x 10^{2}	$(m/c)^{-1}$ min^{-1}	14
kirstructr7	maximum initiation rate constant of *GAL7*	1.928 x 10^{2}	$(m/c)^{-1}$ min^{-1}	12
Kpr10	equilibrium constant for binding of gal4pd to *GAL10*	2.375 x 10^{1}	-	28
Kpr1	equilibrium constant for binding of gal4pd to *GAL1*	8.628	-	26
Kpr2	equilibrium constant for binding of gal4pd to *GAL2*	4.254 x 10^{1}	-	30
Kpr3	equilibrium constant for binding of gal4pd to *GAL3*	5.962	-	33
Kpr7	equilibrium constant for binding of gal4pd to *GAL7*	1.034 x 10^{1}	-	27
Kpr80	equilibrium constant for binding of gal4pd to *GAL80*	3.482 x 10^{1}	-	25
Kq	equilibrium constant for binding of gal80pd to gal4pd-DNA	1.555 x 10^{1}	-	9
krvg3i	dissociation rate constant of Gal_{in}-gal3p complex0	3.493 x 10^{5}	min^{-1}	7
krvg10d	dissociation rate constant of gal10p-gal10p dimer	1.564 x 10^{5}	min^{-1}	39
krvg4dg80d	dissociation rate constant of Gal_{in}-gal3p complex	6.405 x 10^{1}	min^{-1}	29
krvg4d	dissociation rate constant of gal4p-gal4p dimer	1.681 x 10^{2}	min^{-1}	24
krvg7d	dissociation rate constant of gal7p-gal7p dimer	1.860 x 10^{3}	min^{-1}	34
krvg80d	dissociation rate constant of gal80p-gal80p dimer	5.982 x 10^{4}	min^{-1}	46
krvg80g3i	dissociation rate constant of gal80p-gal3p* complex	1.489 x 10^{2}	min^{-1}	6

Supplementary Figure 2

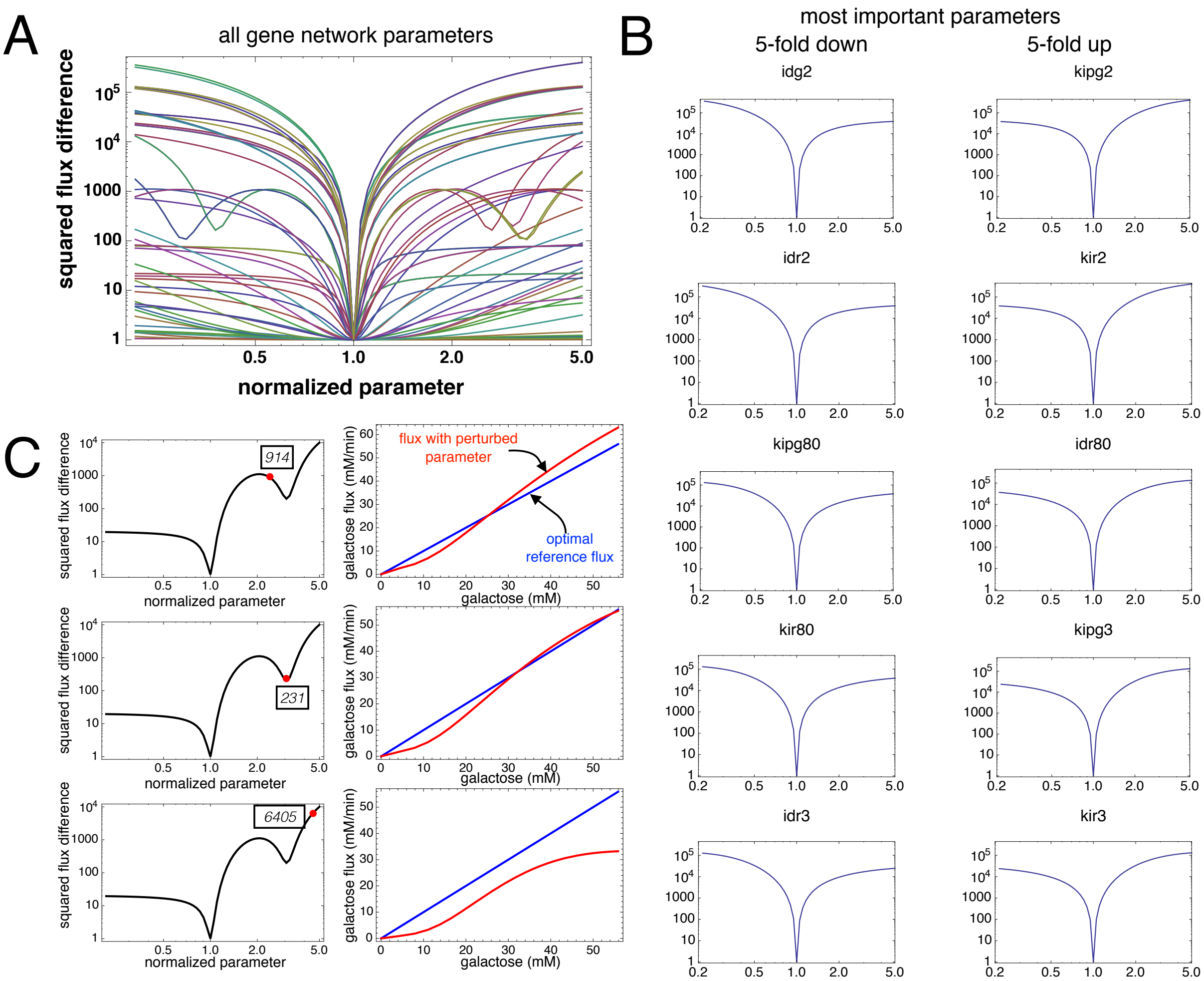

Figure S2: **The effect of perturbation of gene network parameters on metabolic steady state flux.** (A) For every parameter in $\mathbf{p}_g^o$ a reference flux profile for a galactose range of 0 and 50 mM was calculated. The squared distance of this reference flux profile and the flux profile with the perturbed value is plotted for all gene network parameters. (B) The five parameters that have the biggest effect on the galactose flux profile are shown. Left column corresponds to the highest effect when the indicated parameter was perturbed 5-fold down. Right column corresponds to the highest effect when the indicated parameter was perturbed 5-fold up. (C) Explanation of occurrence of multiple minima for some of the gene network parameters, using parameter idg10 as an example. For three values of parameter idg10 (indicated by the red circles), the corresponding galactose flux profiles (red) are shown together with the reference flux profile (blue) in the right columns. The squared flux difference that corresponds with the perturbed value is shown in the black box of every plot.
